# Serum Factors Mediate the Bioenergetic Benefits of Exercise Training and Caloric Restriction

**DOI:** 10.1093/geroni/igaa057.418

**Published:** 2020-12-16

**Authors:** Jenny Gonzalez-Armenta, Barbara Nicklas, Anthony Molina

**Affiliations:** 1 Wake Forest School of Medicine, Winston-Salem, North Carolina, United States; 2 Wake Forest University, Winston-Salem, North Carolina, United States; 3 University of California San Diego, La Jolla, California, United States

## Abstract

Diet and exercise interventions have been shown to improve age-related decline in mitochondrial function. While the systemic benefits of diet and exercise are apparent, the mechanisms underlying these changes are not known. Our lab and others have used blood-based bioenergetic profiling to demonstrate that systemic bioenergetic capacity is related to many aspects of healthy aging, including: gait speed, grip strength, and inflammation. This work suggests a potential role for circulating factors in mediating systemic mitochondrial function. In this study, we developed a high-throughput respirometry assay to examine the effects of circulating factors on mitochondrial function of myoblasts in vitro. We used serum from older, overweight and obese adults who participated in a clinical trial comparing resistance training (RT) and resistance training plus caloric restriction (RT+CR). When combined, both interventions significantly increased serum-mediated basal (42.08 to 50.14 pmol/min, p=0.004), ATP-linked (35.57 to 42.37 pmol/min, p=0.006), and maximal respiration (132.30 to 150.00 pmol/min, p=0.02). With RT, we found significantly increased basal (40.80 to 53.85 pmol/min, p=0.01) and ATP-linked respiration (34.36 to 46.39 pmol/min, p=0.007) and trends for increased maximal respiration (130.09 to 153.24 pmol/min, p=0.10) and spare respiratory capacity (89.30 to 101.38 pmol/min, p=0.07). With RT+CR, there were trends for increased maximal respiration (134.32 to 147.06 pmol/min, p=0.10) and spare respiratory capacity (91.06 to 100.41 pmol/min, p=0.11). Additionally, we found that post-intervention serum-mediated basal and ATP-linked respiration were significantly and positively correlated with physical ability, as reported by SPPB score. Future studies will focus on identifying circulating factors responsible for these changes.

